# Rare Pleomorphic Liposarcoma Presented as Jejunal Obstruction

**DOI:** 10.1155/2023/8040232

**Published:** 2023-06-27

**Authors:** Mohammad Al-Attar, Anup Jnawali, Michelle Yang

**Affiliations:** University of Massachusetts Memorial Health Care, UMass Chan Medical School, Worcester, MA 01605, USA

## Abstract

Pleomorphic liposarcoma (PLS) is typically found in the lower and upper extremities. PLS arising in the gastrointestinal (GI) tract is extremely rare. Here, we reported a case of a 71-year-old female with a history of rectal adenocarcinoma presenting with small bowel obstruction. Small bowel resection was performed and revealed a 7.8 cm transmural mass in the jejunum. Histology reviewed a heterogenous epithelioid malignant tumor with intracytoplasmic fatty droplets scalloping the nucleus consistent with lipoblasts in some cells and others with numerous PAS/diastase+intracytoplasmic eosinophilic globules. Scattered multinucleated giant cells were also present. Mitotic count was up to 80/10 HPFs including some bizarre mitotic figures, and Ki67 proliferation index was approximately 60%. Immunohistochemistry demonstrated that the malignant cells were negative for pancytokeratin, CD117, DOG1, SMA, desmin, MyoD1, ERG1, CD34, CD31, SOX10, Melan A, and S100. INI1 was retained. Beta-catenin showed normal membranous staining. P53 was diffusely positive suggestive of mutant phenotype. Fluorescence in situ hybridization (FISH) assay was negative for MDM2 amplification and DDIT3 rearrangement. The overall morphologic and immunohistochemical features supported a diagnosis of high-grade pleomorphic liposarcoma. Diagnosis of PLS can be challenging due to its rarity in GI tract and lack of specific biomarkers, and histomorphology with identification of lipoblasts remains the gold standard.

## 1. Introduction

Pleomorphic liposarcoma (PLS) is the rarest subtype of liposarcoma accounting for 5-10% of all liposarcomas of soft tissue [1]. About 50% of PLS were found in the lower extremities, followed by upper extremities, axilla, head and neck, and chest region [2–4]. PLS arising in the gastrointestinal (GI) tract is extremely rare with only a handful of cases reported in the literature [5–8].

## 2. Case Presentation

A 70-year-old female had a complex medical history and a rectal adenocarcinoma status postchemoradiation and lower anterior resection in the late 1990s. She subsequently underwent multiple surgical procedures to try to reconnect the colon to rectum, but this was failed due to pelvic scarring. She underwent a revision of the end colostomy in 2000, relocating it from the left to the right side of the abdomen with an ileostomy. Since then, she had intermittent bouts of vomiting and abdominal pain that necessitated multiple hospital visits for possible small bowel obstruction. During the most recent hospital admission for the same complaint in May 2021, her abdominal CT scan revealed the presence of partial small intestinal obstruction caused in part by a parastomal hernia and by a new jejunal mass that was absent on 2020 and 2019 CT scans, indicating a rapidly growing nature of the tumor. A laparoscopic small bowel resection and repair of parastomal hernia were performed.

**Figure 1:**
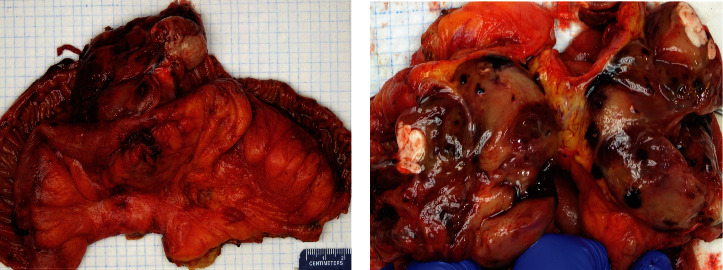
Gross images of the jejunal mass with hemorrhages and necrosis at low (a) and high (b) magnification.

**Figure 2:**
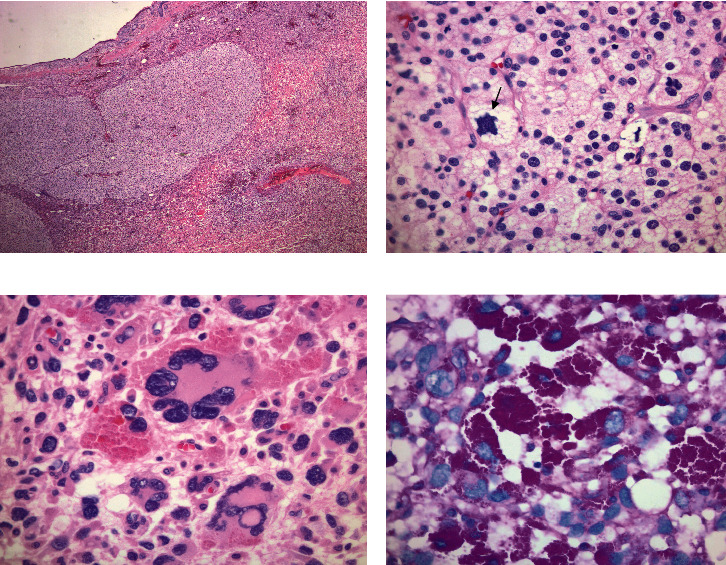
Microscopic features of jejunal pleomorphic liposarcoma: (a) heterogenous population of tumor cells; (b) scattered lipoblasts (arrow); (c) pleomorphic malignant cells with few multinucleated giant cells; (d) PASD positive intracytoplasmic globules. Original magnifications: (a) 20x; (b–d) 400x.

**Figure 3:**
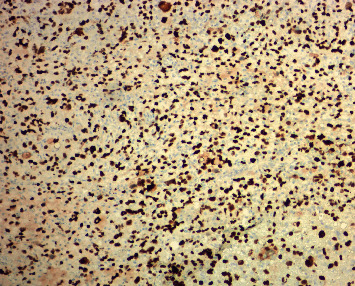
Jejunal pleomorphic liposarcoma with diffusely positive for p53 by immunohistochemistry. Original magnification: 100x.

The jejunal resection specimen revealed a 7.8 × 6.9 × 2.2 cm exophytic mass. On cut surface, the mass lesion showed a transmural heterogenous solid tumor with areas of hemorrhage and necrosis (Figure 1). Multiple lymph nodes, the largest of which measured 1.4 cm in greatest dimension, were also identified in the mesenteric fat. Microscopic examination of the mass showed a high-grade malignant tumor consisting of solid sheets of epithelioid cells (Figure 2(a)), some with intracytoplasmic fatty droplets, scalloped nuclei consistent with lipoblasts (Figure 2(b)), and scattered multinucleated giant cells (Figure 2(c)). Numerous intracytoplasmic PASD-positive hyaline eosinophilic globules were present in some tumor cells (Figure 2(d)). The tumor cells also showed high mitotic count (>80 mitoses per 10 HPFs), bizarre, atypical mitotic figures, and high Ki67 proliferation index (60%). Due to the unusual location and pleomorphic nature of the tumor, a panel of immunohistochemical stains was performed to rule out morphologically similar tumors, such as undifferentiated carcinoma, epithelioid gastrointestinal stromal tumor (GIST), smooth muscle tumors, and malignant melanoma. The tumor cells were negative for multiple lineage specific markers including pancytokeratin AE1/3, CK-OSCAR, EMA, CAM5.2, CD117, DOG1, SMA, desmin, MyoD1, ERG1, CD34, and CD31. HMB45 immunostain revealed rare positive cells. Beta-catenin showed normal membranous staining pattern. P53 was diffusely positive in nuclei suggestive of mutant phenotype (Figure 3). Fluorescence in situ hybridization (FISH) studies revealed the absence of MDM2 amplification or DDIT3 rearrangement. Five lymph nodes identified in the mesenteric fat were negative for metastasis. The overall morphologic and immunohistochemical features supported a diagnosis of high-grade pleomorphic liposarcoma (FNCLCC grade 3 of 3, AJCC pT2 N0) with negative resection margins. No postoperative radiotherapy was documented in our electronic medical record, and the patient remained free of recurrence or metastasis 18 months since the jejunal resection.

## 3. Discussion

Pleomorphic liposarcomas are rare aggressive sarcomas exhibiting local recurrence and metastatic rate of 30-50% and an overall 5-year survival rate of ~60% [9]. In a 57-case series, the median age was 54 years with slightly male predilection [3]. The average size of PLS is about 8 cm, and variable amount of typical lipoblasts can be found in most cases of PLS [10]. FISH analyses for MDM2 amplification and DDIT3 rearrangement were typically necessary to rule out mimickers such as dedifferentiated or myxoid liposarcoma, respectively [11], although MDM2 amplification and FUS-CHOP fusion gene can be occasionally detected in PLS [12, 13]. The pathogenesis and molecular pathways for PLS remain largely unclear. However, p53 overexpression has been reported in the majority of PLS [9, 14].

Radiation therapy has been recognized as a risk factor for developing postradiation sarcoma (PRS), although the incidence of PRS is extremely low ranging from 0.03% to 0.2% with the median period of 11 years post therapy [15]. In our case, the patient had a history of chemoradiation therapy over 30 years ago; therefore, postradiation pleomorphic liposarcoma cannot be completely excluded in our patient. Nonetheless, surgery with curative intent remains the standard management for PRS [15]. Since there are no specific biomarkers to aid the diagnosis of PLS, histomorphology to identify lipoblasts remains the gold standard. The intracytoplasmic hyaline globules (also known as thanatosomes) are a nonspecific morphology since they can be seen in other types of sarcomas, such as malignant peripheral nerve sheath tumor [16], and carcinosarcoma of the gallbladder with chondrosarcomatous differentiation [17]. Adverse prognosis for PLS was associated with nonextremity location, deep-seated lesions, large tumor size ≥ 10 cm, high mitotic rate ≥ 10 per 10 HPFs, necrosis, and epithelioid morphology in univariate analyses [3]. In multivariate analysis, only age ≥ 60 years, central location, tumor size, and mitotic rate were independent prognosticators for an adverse outcome. In other studies, poor prognostic factors for PLS also include primary anatomical site, tumor size, necrosis, epithelioid morphology, and old age [4, 10, 18]. Treatment is focused on local wide excision or resection and adjuvant radiation therapy, which may provide favorable outcome for these patients [3].

## 4. Conclusion

PLS rarely presented an incidental finding after resection of small bowel due to obstruction. Diagnosis of PLS can be challenging due to lack of specific biomarkers, and histomorphology and identification of lipoblasts remain the gold standard.
